# The International Medical Annual and Practitioner’s Index

**Published:** 1898-04

**Authors:** 


THE INTERNATIONAL MEDICAL ANNUAL AND PRACTITIONER'S INDEX. A work of Reference for Medical Practitioners. 1898. Sixteenth year. New York: E. B. Treat &

Co., 241 and 243 West Twenty-third Street, Chicago. Price, $3.00.

This book is regularly brought to the notice of our subscribers every year It is an epitome, a summary, of all the advancement made in medicine during the past year. Whoever possesses himself of it has a fine retrospect of medical progress, and if he be so fortunate as to have the whole series of sixteen volumes he has at his command the history of medical thought and its changes during the past sixteen years. To facilitate this reference the book is supplied with an excellent alphabetical index which in the present volume occupies twenty-four pages double column. Thus, if it be desired to know what have been the changes in idea concerning Typhoid Fever, we have but to turn to that word in the index in each of the volumes, and there we have before us a superior condensed statement of everything that has been discovered about it during the year for which the volume consulted was published.

By taking *all* the volumes for sixteen years and turning to Typhoid Fever in each one, and reading them in chronological order, we have an excellent retrospect of the subject for sixteen years. This certainly is commendation enough to secure for these volumes the regard of the whole medical profession. Even those who are too much taxed for time to enable them to indulge their taste for study and investigation will find in these volumes the information they seek in the most complete, yet concise form, and obtainable with the least loss of time.

Looking through the present issue of this admirable work, we notice in the first part comment on drugs. The drugs are in alphabetical order, and one is assisted in getting at them by the thorough index. Then there is a chapter on electrical improvements, illustrated. There is a short chapter on hypnotism and mental suggestion by Dr. Tuckey.

In part II on new treatment we notice articles on abdominal surgery, abortion, albuminuria, amenorrhœa, amputations, aneurism, anthrax, appendicitis, aphonia, arthritis, and asthma. These subjects we take at random from the letter A, the better to show the scope of the book.

In the article on albuminuria, cases are reported of the cure of albuminuria by puncturing the kidney capsule.

Under "dermatitis" is shown a fine plate, illustrating the production of dermatitis by the action of the X-rays. It is indeed a proving of the X-ray.

In the article on diphtheria, photographs by the X-ray are shown of the throat of a child to show the position of the tube in intubation.

Curious cases of the dislocation of the knee are reported with wood-cut illustrations.

Elaborate surgical procedures for the treatment of the ear are shown, especially for the restoration of the external drum. The treatment of fractures of the leg so as to permit of walking during the progress of bony union is described, and fine plates, illustrating the method of managing such cases are given. Congenital dislocation of the hip is liberally illustrated by plates, and methods of treatment are explained.

The comparatively new operations of suture of the intestines are well treated and finely illustrated, and the latest surgical procedures on the kidney are also shown.

Pott's Disease, with methods of correcting the deformity, are described and profusely illustrated with photographic prints.

All these are exceedingly interesting, of great practical value to every enlightened physician, and need to be well known if the physician wishes to be up-to-date, and not falling behind in the progress of this wonderful age.

